# Medical Holidays

**Published:** 1898-09-10

**Authors:** 


### The Hospital

A Journal of

The Medical Sciences and Hospital Administration.

Vol. XXIV.—No. 624.] Edited by Sir Henry Burdett, K.C.B., and Solomon C. Smith, M.D., M.R.C.P. [Sept. 10, 1898.

### Medical Holidays.

Thackeray, in one of the wisest of his ballads, tells us that

> " True philosophers, methinks,
> Who love all sorts of natural beauties,
> Should love good victuals and good drinks."

And, if this be so, it is inevitable that the medical philosopher, whose time is mainly spent in preserving or in restoring for other people the natural beauties of health, should love the proceedings by which he is enabled to extend this beneficent action to his own proper person. Few things are more curious than the extent to which holidays are now thought necessary to the welfare and working capacity of every class of the community. Fifty years ago they were at least exceptional, and we doubt whether they were ever taken by any members of the medical profession other than the most highly placed consultants. It used to be said of Sir Henry Holland, who every year left his practice for two months, and sped to the most remote part of the earth's surface which could be reached and returned from in the time, that his holidays were of advantage to his professional brethren by giving him opportunities for writing books so delightful that those who read them could make holiday at home. A country doctor, as a rule, remained in harness from one year's end to another, and we well remember being told, five and thirty years ago, by a surgeon in the Midlands, who went all over his county as an operator and consultant, that he rejoiced in a summons to France to see a patient who had been taken ill while travelling there, because, although he was sixty-two years old, he had never seen the sea. Such a statement would now appear incredible, but it may still be doubted whether the universality of holiday making has been attended with any very great development of the art of making holiday profitably and wisely. Men do, indeed, "run to and fro," but is it also true that knowledge, in so far at least as the art of running to and fro is concerned, has increased in any corresponding ratio? That the facilities have increased every day's experience may tell us, but these lend themselves to abuse as easily as to use, to loss as easily as to gain. The medical man's holiday should surely be an example to those whom it is his business to guide in the paths which lead to health, and should not be what it often becomes, a mere period of time diverted from profitable and useful to unprofitable and useless occupations.

A holiday, in the first place, should be a period of comparative rest, and, so far, of both physical and mental recuperation, or, to put the matter more accurately, a period in which both nerve cells and muscular fibre, and the organs formed by their aggregation, should enjoy a repose which they may turn to account in the direction of increased efficiency. The ideal is not attained, for example, by a form of holiday which involves any great increase of physical exertion. The doctor whose bodily exercise, for eleven months of the year, is taken in a brougham, or in ascending and descending staircases, may derive unmixed benefit from the crisp and invigorating air of mountainous districts, and even from some moderate extension of his accustomed exercise. He will not derive benefit from daily over-fatigue but will be more likely to render himself ill from excess of tissue waste, and from the strain thrown upon his excretory glands by the work of removing it. In the same way many of the most popular health resorts are notorious for the indigestion which they constantly inflict upon visitors. A more stimulating atmosphere than that of home leads at once to a great increase of appetite for food; and this, if indulged without much circumspection, works its revenge in prompt fashion and in familiar ways. The hurry, too, of modern life, bad enough and unwholesome enough even when it is inevitable, is worse still when it is allowed to invade periods of time which should be kept sacred from its influence. In its very nature it is essentially antagonistic to the idea of a holiday, an idea which should rest firmly upon that of refreshment after labour. To rush to this or that sight before luncheon, and to another between luncheon and dinner; to cover an additional and unnecessary ten or twenty miles with the bicycle in order to reach a more distant sleeping-place; or to curtail the hours of sleep in order to crowd more achievement into the coming day; all these are practices which are common enough, but which, as a moment's reflection will show, are likely to defeat the very objects with which they are professedly undertaken, and to accentuate wear and tear in place of affording repose. It is for this reason, in all probability, that many customarily moderate smokers seek to diminish the discomforts of holiday making by the excessive use of tobacco, and try to conceal from themselves, by its influence, the consequences to which they voluntarily submit. The remedy is as delusive as the mischief, which it may for a time obscure, is certain. Let it once be understood that the object of a holiday is to rest both body and mind, and then the time which can be spared for the purpose is likely to be wisely and well employed.

